# Supplementary material for: A neural circuit model of decision uncertainty and change-of-mind
Source: Nat Commun. 2019 May 23;10:2287. doi: 10.1038/s41467-019-10316-8 (PMC6533317; doi:10.1038/s41467-019-10316-8)
Supplement: Supplementary file 1 — Supplementary Information [file 41467_2019_10316_MOESM1_ESM.pdf]

A Neural Circuit Model of Decision Uncertainty and Change-of-Mind

Supplementary Information

Atiya et al.

Supplementary Note 1: Justification of our modelling choices

a) Tonic activity**:** In the uncertainty-encoding population, the tonic activity (i.e. elevated baseline background activity) provides a mechanism by which inhibitory input/signal can be transmitted. In particular, if the receiving neuronal population firing rate was to be silent (i.e. no tonic activity), then only excitatory input can be transmitted. Specifically, in such cases, inhibitory input cannot be transmitted due to the 'flooring' effect (i.e. the neuronal firing rate cannot be negative).

b) Inhibitory-excitatory pair of populations in uncertainty-monitoring module**:** The pair of excitatory and inhibitory neural populations in the uncertainty-monitoring module resembles the simplest plausible computational model of a cortical column[1] – we assume that uncertainty is encoded in the cortex, e.g. the frontal cortex[2–4]. Second, and importantly, the key computational role of this inhibitory population is not only to restrain but to modulate the activity levels of the uncertainty-encoding population, and hence influencing the encoding of decision uncertainty (see Results section).

c) Top-down (dis)inhibition: Used in our model, such an inhibitory mechanism has been proposed to originate from various brain regions, e.g. involving the superior colliculus and basal ganglia. For example, the threshold crossing (response threshold in our model, which triggers top-down inhibition) could be detected by the superior colliculus via basal ganglia[5,6]. More complex gating pathways in the brain, including disinhibitory circuits, have been proposed that also involve subcortical structures, such as the basal ganglia and thalamus[7]. It should be noted that providing an explicit account of such complex neural circuit dynamics is beyond the scope of this work. As a proxy to modelling such complex and extended neural networks, we have instead modelled just the onset and offset of the top-down inhibition. The onset of this top-down inhibition is assumed to have been learned e.g. through the basal ganglia[8], which is mediated through neuromodulators.

Supplementary Note 2: Multi-stage decision paradigm

The multi-stage decision paradigm we simulated is a special case of sequential decision-making[9]. Specifically, two coupled decisions have to be correct in order to receive a reward. The time delay from the first choice to the next stimulus onset (response-stimulus interval, RSI) is sampled from a truncated exponential distribution (range 0.3–1.0 s; mean 0.57 s). When simulating this paradigm, we have reset the uncertainty bias upon the completion of every pair of coupled trials. Thus, our implementation of the paradigm would not be affected by the RSI. Our stored uncertainty bias could perhaps be allowed to decay over time, for instance, similar to our previous work[10]. However, to the best of our knowledge, this multi-stage decision study[9] is the only published work that links decision confidence to response times with sequential dependency, and we defer such speculation to further experimental evidence.

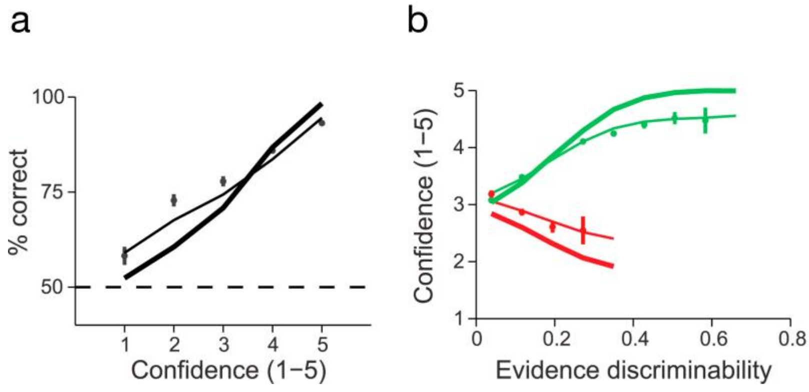

Supplementary Figure 1. Common characteristics of decision confidence. (**a-b**) Thick and thin lines are two statistical model fits of an experimental study. Combined data of all subjects (n = 5) (**a**) Choice accuracy as a function of decision confidence. Monotonic increase in accuracy (% correct) with increasing confidence level. (**b**) < (or sometimes called X) pattern of decision confidence with respect to increasing evidence discriminability (quality) i.e. decreasing task difficulty. Decreasing (increasing) confidence for error (correct) choices with increasing stimulus strength. Green: Correct choices; red: error choices. Reused from[11] with permission. Similar pattern reported in[2,12,13]. Error bars show 95% confidence interval of the mean

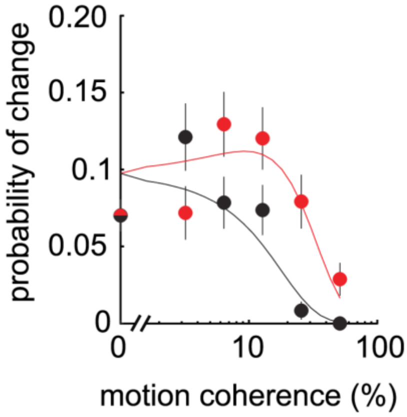

Supplementary Figure 2. Common characteristics of change-of-mind. Correct changes-of-mind (red) are generally more frequent than error changes-of-mind (black) and peak at an intermediate task difficulty level (the task difficulty is the % of motion coherence in random dot kinematogram of a motion discrimination task) before decreasing. Data fitted with an extended drift-diffusion model. Reused from[14] with permission. Error bars are s.e.m.

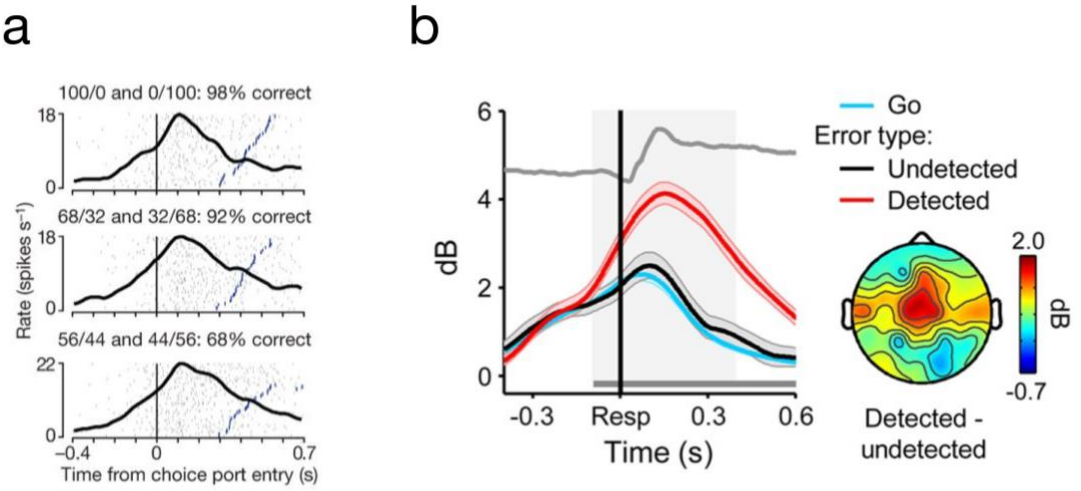

Supplementary Figure 3. Phasic neural activities of decision uncertainty. (**a**) Opt-out waiting task in which the animal can opt-out for less-but-sure reward. The time it takes the animal to opt-out, a measure of decision uncertainty, correlates with the single neuronal firing rate in the orbitofrontal cortex (OFC). Each row corresponds to a stimulus difficulty (top: easiest; middle: easy; bottom: difficult). Note the small but non-zero firing activity at baseline, and the slightly higher peak of the firing rate with lower accuracy (bottom row). Reused from[2] with permission. (**b**) A Go/No-Go decision-making task in which participants can make mistakes.

Averaged EEG responses (theta band) (left) from the frontoparietal cortex (right) is associated with participants detecting their errors. Scalp topography shows the distribution of error detection effect (signal-to-noise ratio is shown in dB). Reused from[3] under the Creative Commons Attribution 4.0 International Public License.

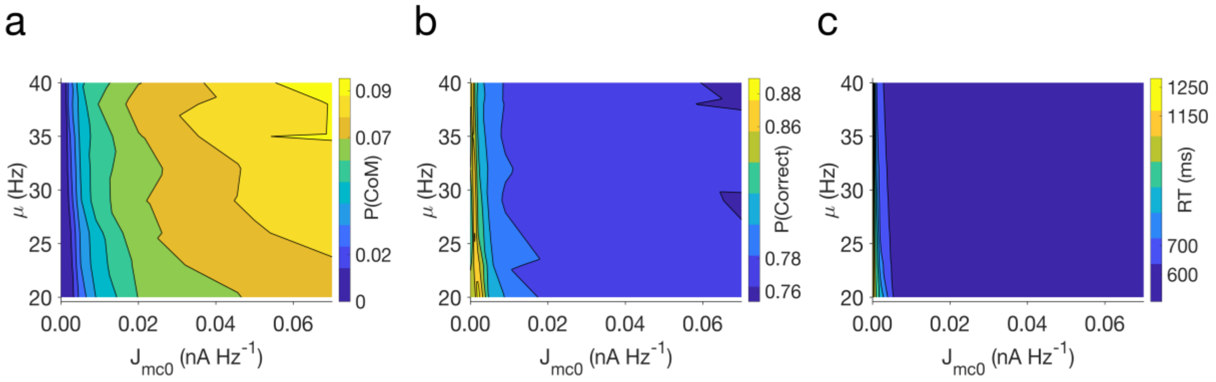

Supplementary Figure 4. Excitatory feedback determines model performance. All changes in parameters are simulated with 8000 trials under one condition ($\varepsilon = 6.4\%$). μ: tonic input to the uncertainty-encoding neural population; J_{mc0}: excitatory feedback strength from uncertainty-monitoring module to sensorimotor module. Effects across other evidence quality levels can be inferred from the main simulation results (see Fig. 1 and Fig. 4 in the main manuscript). (**a**) Probability of changes-of-mind (CoM), P(CoM). P(CoM) is increased with increasing excitatory feedback strength. (**b**) Accuracy (probability of correct, P(Correct)) increases as the excitatory feedback strength from the uncertainty-monitoring module to sensorimotor module is increased. Saturation at around 77% accuracy due to reaching the fixed decision threshold (35.5Hz, see Methods). (**c**) Faster response times (RTs) are observed when excitatory feedback strength is increased. Saturation of RTs at 600ms due to reaching the fixed decision threshold (35.5 Hz, see Methods).

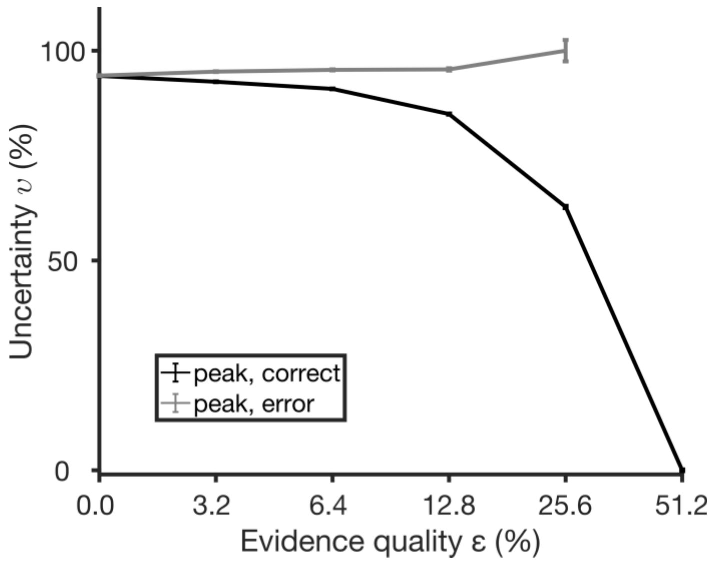

Supplementary Figure 5. Decision uncertainty depends on evidence quality. 8000 trials per condition of evidence quality. List of parameter values used in this simulation can be found in Supplementary Table 1, with the exception of the excitatory feedback strength (J_{mc0}) set to 0. Model can encode decision uncertainty without the feedback loop but with no change-of-mind (see Supplementary Figure 4). Error bars are s.e.m.

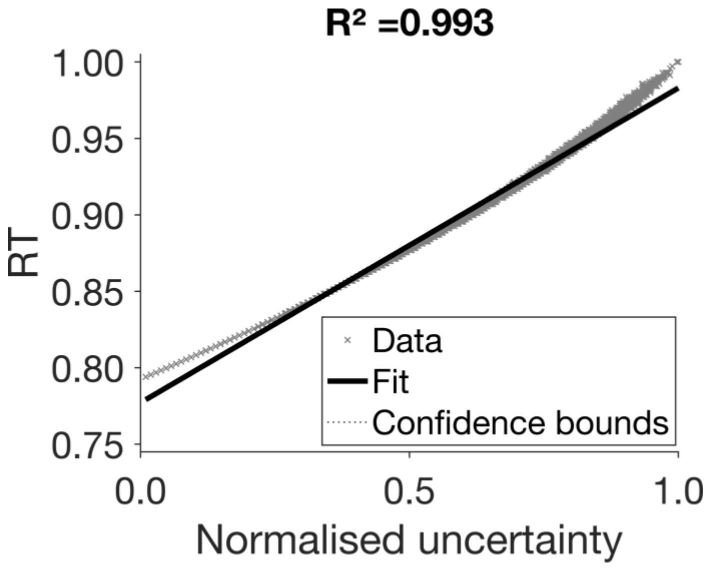

Supplementary Figure 6. Decision uncertainty is strongly linked to response time. Data points (blue) of individual response times (RTs) were obtained from 8000 simulated trials per evidence quality level (48,000 trials). Data indiscriminately consisted of correct, error, CoM and non-CoM trials. Fit (red line) was performed using a linear regression of the uncertainty level as a function of RTs. The two variables (uncertainty level and RT) have a very high (Pearson's) correlation coefficient of 0.85 (p-value = 0) (not shown). Decision uncertainty was calculated using the maximal activity level (similar results with area under the curve method – not shown) (see Methods for further detail). High evidence quality trials that resulted in insignificant change (i.e. <1 Hz) in decision uncertainty levels were excluded from the analysis (6042 trials).

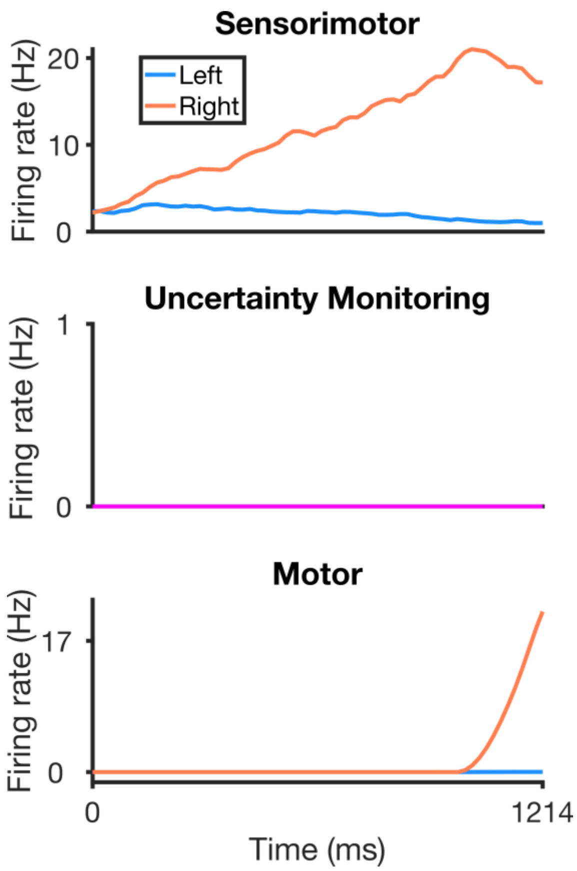

Supplementary Figure 7. Easy trial: a sample activity timecourse. Sample timecourse of firing rates in sensorimotor module (top panel), uncertainty-encoding population (middle panel), motor module (bottom panel). Easiest difficulty ($\varepsilon = 51.2$). When the motor activity

crosses the 17 Hz threshold, the motor output is assumed to reach a choice target. Due to faster ramping up of activity, the response threshold in the sensorimotor population (35.5 Hz) is crossed before the temporal integration of uncertainty-encoding population begins. Trial completion time (time target threshold was reached) at 1214 ms. Neural population firing rates were calculated by averaging over a time window of 50 ms, slided with a time step of 5 ms.

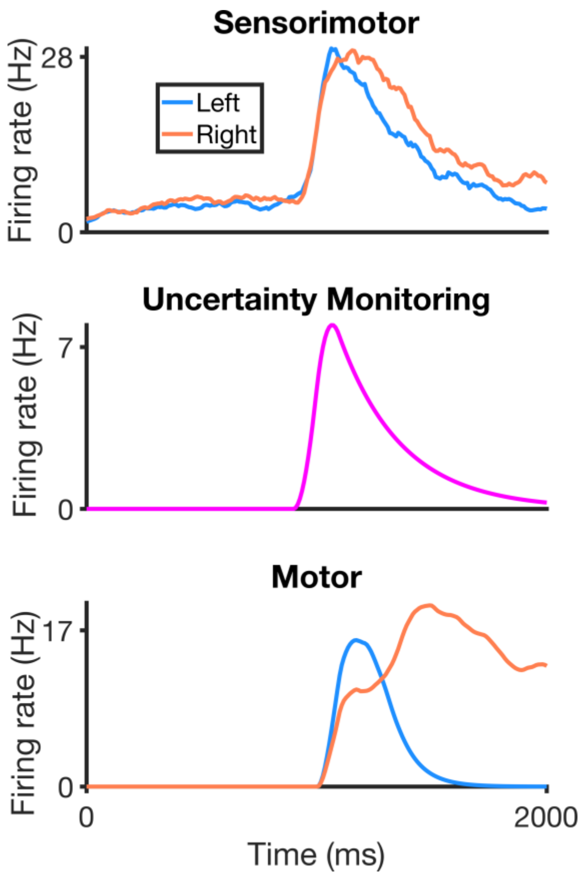

Supplementary Figure 8. A sample neural activity timecourse during change-of-mind. Sample timecourse of firing rates in sensorimotor module (top panel), uncertainty-encoding population (middle panel), motor module (bottom panel). ($\varepsilon = 3.2$) exhibiting change-of-mind. Despite the small difference in the dominance of activity in the sensorimotor module, the motor populations continue to integrate over time, amplifying this difference. Trial completion time (time target threshold was reached) at 1637 ms. Neural population firing rates were calculated by averaging over a time window of 50 ms, slided with a time step of 5 ms. Integration is shown

until 2000ms to reveal the full dynamics (the phasic nature) of the uncertainty-encoding population activity (middle panel).

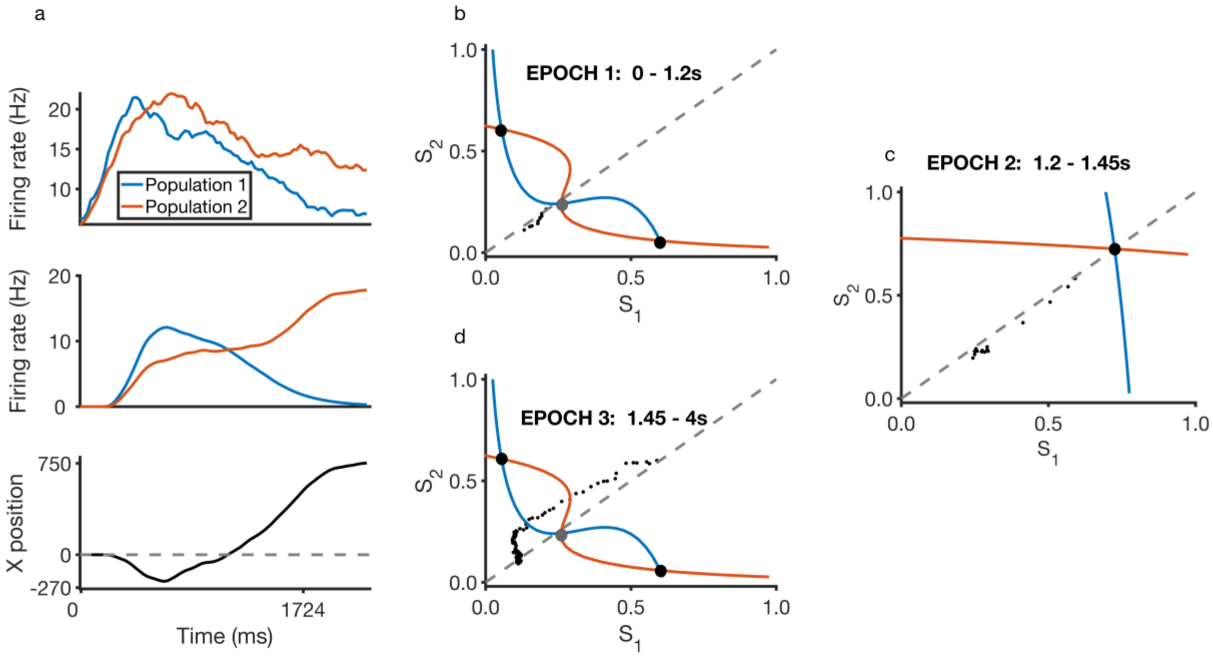

Supplementary Figure 9. Dynamics of a sample change-of-mind trial. (**a**) Timecourse of firing rates in sensorimotor module (top panel), motor module (middle panel) and motor trajectory (bottom panel) with bias input (evidence quality) $\varepsilon = 3.2$ (favouring population/choice 2/Right). Left (blue line) and right (orange line) populations compete after stimulus onset. As motor starts moving in one direction (without reaching the target), a reversal of neural activity dominance in sensorimotor module and motor module occurs. This leads to a change-of-mind. (**b**) Immediately upon stimulus onset with evidence quality $\varepsilon = 3.2$ (favouring choice 2/Right), the sensorimotor population activity trajectory (black dotted line) in phase space starts to deviate from the phase plane diagonal. Black filled circles: stable steady states representing the two choices i.e. choice attractors; grey filled circle: saddle-like unstable steady state. Refer to the main manuscript and previous work[15] for details regarding the content of the phase plane (e.g. nullclines) (**c**) During the middle epoch of the trial, there is a large excitatory

feedback from the uncertainty-monitoring monitoring module, such that the phase plane of the sensorimotor module reconfigures, and a new choice-neutral stable steady state appears which aids the initially losing neural population (population 2). The trajectory is now drawn towards this stable steady state, moving back towards the phase plane diagonal. (**d**) During the later epoch of the trial, both sensorimotor populations receive lesser excitatory feedback from the uncertainty-monitoring module, resulting in the phase plane reverting to a similar configuration during the one seen in an early epoch of the trial. It should be noted that a decision is still made by the differential activity amplified by the motor populations, as they continue to integrate excitatory input from the sensorimotor module. Neural population firing rates were calculated by averaging over a time window of 50 ms, slided with a time step of 5 ms.

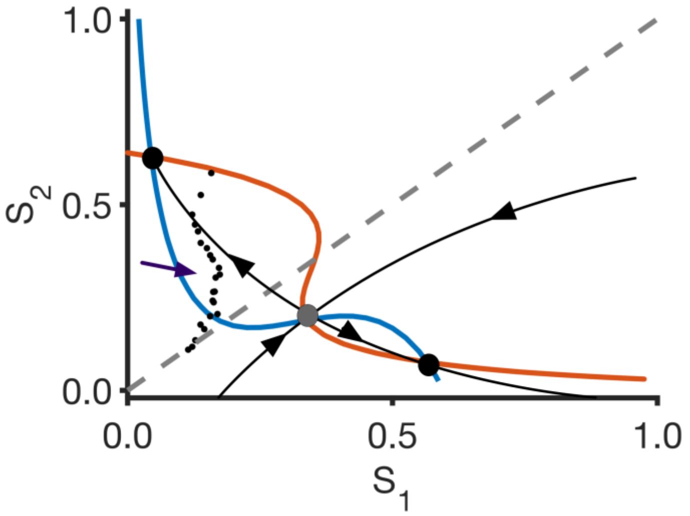

Supplementary Figure 10. Phase-plane asymmetry in an easy task. Phase space of the sensorimotor module after stimulus onset with evidence quality $\varepsilon = 25.6$. Most of the time, the trajectory will move directly towards the favoured stable steady state i.e. in this case, the attractor representing choice 2/Right. In rare cases where the central choice-neutral stable steady state transiently emerges for such highly biased input, the network would very likely continue its path towards the favoured ('stronger') choice attractor. This is due to the larger

basin of attraction of the favoured attractor[15]. The trajectory shown (black dotted line) from stimulus onset to response confirms the dominance of the stable choice attractor albeit the presence of a ‘kink’ (purple arrow) indicating the transient presence of the central attractor briefly perturbing the trajectory.

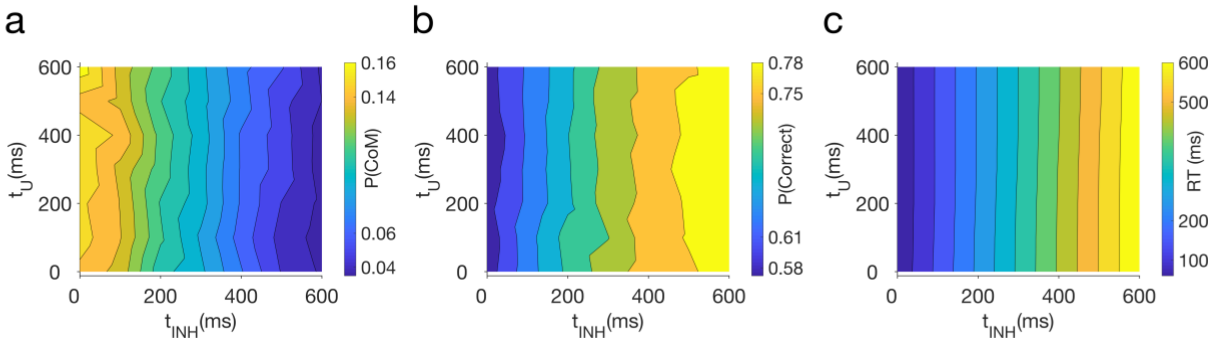

Supplementary Figure 11. Activation time of top-down inhibition affects model performance. All changes in parameters are simulated with 8000 trials under one condition ($\varepsilon = 6.4\%$). Effects across other difficulty levels can be inferred from the main simulation results (see Fig. 1 and Fig. 4 in manuscript). t_U is the time delay from stimulus onset to activation onset (by removing the top-down inhibition) of the uncertainty-encoding population, while t_{INH} is that for the inhibitory population in the uncertainty-monitoring module. Probability of changes-of-mind P(CoM) decreases (a), accuracy increases (b) , and response time (RT) is slower (c), as the delay t_{INH} is increased.

Parameter	Value	Reference, remarks
$J_{N,ii}$	0. 244*nA*	Modified from [15]
$J_{N,ij}$	0.0497 *nA*	[15]
I_0	0.3255 *nA*	[15]
J_{mc0}	0.009 *nA* Hz^{-1}	Fit to experimental data
$J_{A,ext}$	0.00052 *nA* Hz^{-1}	[15]
μ_0	30 Hz	[15]
$J_{N,\mathrm{U\_inh}}$	0.5 Hz Hz^{-1}	Fit to experimental data
$J_{N,LR}$	2 *nA*	Modified from [16]
$J_{N,RL}$	2 *nA*	Modified from [16]
τ_h	*50 ms*	Fit to experimental data
τ_{mc}	*150 ms*	Fit to experimental data
τ_{s}	*100 ms*	[15]
a	*270 (V nC)*$^{-1}$	[15]
b	*108 Hz*	[15]
d	*0.154 s*	[15]
t_{U}	*500 ms*	Fit to experimental data
t_{inh}	*500 ms*	Fit to experimental data

Supplementary Table 1: Summary of model parameter values. See main text for parameter description.

Supplementary References:

1. Wilson, H. R. & Cowan, J. D. Excitatory and inhibitory interactions in localized populations of model neurons. *Biophys. J.* **12**, 1–24 (1972).
2. Kepecs, A., Uchida, N., Zariwala, H. a & Mainen, Z. F. Neural correlates, computation and behavioural impact of decision confidence. *Nature* **455**, 227–231 (2008).
3. Murphy, P. R., Robertson, I. H., Harty, S. & O'Connell, R. G. Neural evidence accumulation persists after choice to inform metacognitive judgments. *Elife* **4**, 1–23 (2015).
4. Fleming, S. M., van der Putten, E. J. & Daw, N. D. Neural mediators of changes of mind about perceptual decisions. *Nat. Neurosci.* (2018). doi:10.1038/s41593-018-0104-6
5. Lo, C. & Wang, X. Cortico – basal ganglia circuit mechanism for a decision threshold in reaction time tasks. **9**, 956–963 (2006).
6. Crapse, T. B. & Sommer, M. A. Frontal Eye Field Neurons with Spatial Representations Predicted by Their Subcortical Input. **29**, 5308–5318 (2009).
7. Jaramillo, J., Mejias, J. F. & Wang, X. Engagement of pulvino-cortical feedforward and feedback pathways in cognitive computations. (2018).
8. Hazy, T. E., Frank, M. J. & Reilly, R. C. O. Towards an executive without a homunculus : computational models of the prefrontal cortex / basal ganglia system. 1601–1613 (2007). doi:10.1098/rstb.2007.2055
9. van den Berg, R., Zylberberg, A., Kiani, R., Shadlen, M. N. & Wolpert, D. M. Confidence Is the Bridge between Multi-stage Decisions. *Curr. Biol.* **26**, 3157–3168 (2016).
10. Gao, J., Wong-Lin, K., Holmes, P., Simen, P. & Cohen, J. D. Sequential effects in two-choice reaction time tasks: decomposition and synthesis of mechanisms. *Neural Comput.* **21**, 2407–2436 (2009).
11. Sanders, J. I., Hangya, B. & Kepecs, A. Signatures of a Statistical Computation in the Human Sense of Confidence. *Neuron* **90**, 499–506 (2016).
12. Lak, A. *et al.* Orbitofrontal cortex is required for optimal waiting based on decision confidence. *Neuron* **84**, 190–201 (2014).
13. Fleming, S. M. & Daw, N. D. Self-evaluation of decision-making: A general Bayesian framework for metacognitive computation. *Psychol. Rev.* **124**, 91–114 (2017).
14. Resulaj, A., Kiani, R., Wolpert, D. M. & Shadlen, M. N. Changes of mind in decision-making. *Nature* **461**, 263–266 (2009).
15. Wong, K.-F. & Wang, X.-J. A Recurrent Network Mechanism of Time Integration in Perceptual Decisions. *J. Neurosci.* **26**, 1314–1328 (2006).
16. Gonçalves, P. J., Arrenberg, A. B., Hablitzel, B., Baier, H. & Machens, C. K. Optogenetic perturbations reveal the dynamics of an oculomotor integrator. *Front. Neural Circuits* **8**, 1–22 (2014).
